# Hipertensão Arterial e Ácido Úrico Sérico em Idosos - Estudo SEPHAR III

**DOI:** 10.36660/abc.20200004

**Published:** 2021-08-09

**Authors:** Roxana Buzas, Vlad-Sabin Ivan, Oana-Florentina Gheorghe-Fronea, Adina Flavia Morgovan, Melania Ardelean, Nicolae Albulescu, Maria Dorobantu, Daniel Florin Lighezan

**Affiliations:** 1 University of Medicine and Pharmacy Victor Babes Timisoara Department of Internal Medicine Timisoara Romênia University of Medicine and Pharmacy Victor Babes Timisoara - Department of Internal Medicine, Timisoara – Romênia.; 2 University of Medicine and Pharmacy Cardio-Thoracic Pathology Department Bucuresti Romênia Carol Davila University of Medicine and Pharmacy - Cardio-Thoracic Pathology Department, Bucuresti – Romênia.

**Keywords:** Hipertensão, Ácido Úrico, Hiperuricemia, Doenças Cardiovasculares, Filtração Glomerular, Efeito-Idade

## Abstract

**Fundamento:**

A hiperuricemia é um achado frequente em pacientes com hipertensão arterial e há evidências cada vez maiores de que essa entidade seja também um fator de risco para doença cardiovascular.

**Objetivos:**

No contexto da população em processo de envelhecimento, este estudo tem o objetivo de avaliar níveis de ácido úrico sérico e a prevalência e o controle da hipertensão arterial em um subgrupo da população de adultos romenos (>65 anos), em relação à influência da idade nesses parâmetros.

**Métodos:**

A amostra do estudo consiste em 1920 adultos incluídos na pesquisa SEPHAR III, dos quais 447 eram pacientes idosos (>65 anos de idade). Durante as duas visitas do estudo, três aferições de pressão arterial (PA) foram realizadas em intervalos de 1 minuto, e foram realizadas medições de níveis de ácido úrico sérico, função renal por taxa de filtração glomerular, pressão arterial e espessura íntima-média. A hipertensão e os controles foram definidos de acordo com as diretrizes atuais. A avaliação da espessura íntima-média foi determinada pela avaliação por ultrassom Doppler modo B. Um nível de significância p < 0,05 foi adotado para a análise estatística.

**Resultados:**

Pacientes adultos tinham níveis de ácido úrico sérico significativamente mais baixos, se comparados a pacientes idosos, independentemente dos níveis de taxa de filtração glomerular. Pacientes adultos tinham níveis de espessura íntima-média, comparados a pacientes idosos.

**Conclusão:**

De forma semelhante às pesquisas anteriores, neste estudo, a idade representou um dos fatores contribuintes ao nível aumentado de ácido úrico sérico. Também foi obtido um aumento da prevalência da hipertensão arterial com a idade, com um mau controle da pressão arterial.

## Introdução

A expectativa de vida continua a aumentar em países desenvolvidos em todo o mundo, levando a um constante aumento da representação de adultos mais velhos (pessoas com mais de 65 anos de idade) dentro da população.^1^

De acordo com o oitavo relatório do *Joint National Committee* (JNC 8), aproximadamente 970 milhões de pessoas em todo o mundo têm pressão arterial alta. Estima-se que, até 2025, 1,56 bilhões de adultos estarão convivendo com a hipertensão arterial (HT). A etiologia da HT essencial ainda é desconhecida, e sua patogênese inclui vários fatores genéticos e ambientais. Mais de dois terços dos indivíduos com idade superior a 65 anos sofrem de HT, de acordo com o sétimo relatório do *Joint National Committee* (JNC-7).^2^ Vários estudos epidemiológicos indicam que a incidência de HT e das doenças cardiovasculares associadas é mais alta na população mais velha que na população jovem.^2^^,^^3^ Um estudo de sua prevalência e controle entre adultos nos Estados Unidos no período de 1999 a 2004 demonstrou que a prevalência de HT mais que dobrou entre os idosos, em comparação aos jovens. Mesmo que a crença geral seja de que a HT é um distúrbio do envelhecimento, nos últimos anos, a população de meia idade parece ter tido um aumento na incidência da hipertensão arterial.

Por outro lado, a hiperuricemia é mais comum, e há vários estudos mostrando que os níveis de ácido úrico sérico estão associados a um aumento na prevalência da hipertensão (HT) que também contribui para falta do controle ideal de pressão arterial (PA).^4^.

O SEPHAR (*Study for the Evaluation of Prevalence of Hypertension and Cardiovascular Risk in Romania* - Estudo para a Avaliação da Prevalência da Hipertensão e do Risco Cardiovascular na Romênia) é um projeto que tem o objetivo de avaliar a prevalência da HT e outros fatores relacionados, incluindo o ácido úrico sérico. Até hoje houve três estudos SEPHAR separados com intervalo de vários anos, sendo que o SEPHAR II, realizado em 2012, foi o primeiro a avaliar os níveis de ácido úrico sérico, que também correlacionaram os níveis de AUS com a espessura íntima-média, a função renal, e o risco cardiovascular. Continuando com o SEPHAR III em 2016 que ofereceram dados adicionais sobre os níveis de AUS e sua relação com a prevalência de HT na Romênia, vários outros índices foram usados, tais como TFG e parâmetros ecocardiográficos. O SEPHAR III foi desenhado como uma pesquisa transversal que caracteriza dados para a população adulta na Romênia quanto à prevalência de HT, controle, e agentes hipertensivos.^5^^,^^6^.

Este estudo tem o objetivo de avaliar níveis de ácido úrico sérico (AUS) e a prevalência e o controle de EIM e HT em um grupo da população de adultos romenos, em relação à população idosa.

## Materiais e Métodos

Uma van médica batizada de Ônibus SEPHAR foi utilizada para realizar duas visitas com 4 dias de diferença. No total, 1920 adultos romenos foram cadastrados na pesquisa SEPHAR III (média de idade de 48,63 anos, 52,76% do sexo feminino) dos quais 447 eram pacientes idosos (23,28% com idade igual ou superior a 65 anos). Os pacientes foram examinados e 3 aferições de PA foram realizadas, na posição sentada, de acordo com as atuais Diretrizes Europeias para monitoramento de PA, em intervalos de um minuto. Durante cada visita do estudo, 3 aferições de PA foram registradas, com um dispositivo automático de aferição de PA (OMRON M6). O manguito foi ajustado de acordo com a circunferência do braço, e todas as aferições foram realizadas no mesmo braço que apresentou os maiores valores de PA durante a visita inicial.

Uma pressão arterial sistólica (PAS) maior que 140 mmHg e/ou uma pressão arterial diastólica (PAD) acima de 90 mmHG em ambas as visitas foi considerada HT, utilizando-se a média do segundo e do terceiro valores de PA de cada visita. A primeira PA de cada visita não foi levada em consideração para análise futura. Além disso, a HT conhecida e tratada, com PA controlada ou não controlada, durante as duas semanas anteriores, também foi levada em consideração.

Para que um sujeito tenha PA controlada, as diretrizes ESH-ESC de 2018 sobre a hipertensão foram utilizadas, definindo como controle de PA para pacientes hipertensos com pelo menos 2 semanas de tratamento prévio, PAS e PAD menores que 140 mmHg e 90 mmHg respectivamente.

A análise da amostra sanguínea que incluiu o AUS mencionado foi realizada durante a segunda visita, com o paciente sendo informado na primeira visita de que era necessário um período de jejum de pelo menos 8 horas. Os níveis de AUS foram analisados com um equipamento analisador COBAS 6000 com reagentes uricase/peroxidase, com valores normais dados entre 2,4 e 5,70mg/dl, para mulheres, e 3,40 a 7,00mg/dl para homens. A hiperuricemia foi diagnosticada quando foram identificadas faixas normais. Para a avaliação da função renal, os valores da fórmula de Modificação Dietética na Doença Renal (MDRD) e da fórmula Colaboração de Epidemiologia de Doença Renal Crônica (CKD-EPI) foram calculados e utilizados nas análises estatísticas.

Utilizou-se um aparelho para ecocardiograma portátil (modelo General Electric Vivid Q), que calculou a espessura íntima-média (EIM) de cada parede distal da artéria carótida comum 1 cm abaixo do bulbo da carótida. A EIM foi medida usando-se uma sonda linear com frequência ajustável entre 7,5 e 10 MHz.

O Comitê de Ética da Faculdade de Medicina e Farmácia Carol Davila, em Bucareste na Romênia, aprovou o estudo em total conformidade com a Declaração de Helsinki, e foi obtido o consentimento informado por escrito de todos os participantes antes da realização de qualquer exame.

### Análise estatística

Os resultados de variáveis específicas foram apresentados utilizando-se contagens com porcentagens para dados categóricos, e estatísticas descritivas (média, desvio padrão) para dados contínuos. As diferenças entre médias e variáveis contínuas foram analisadas utilizando-se testes t para amostras independentes, enquanto testes Qui-quadrado foram usados para examinar diferenças entre variáveis categóricas. Considerando-se que a normalidade do tamanho da amostra foi presumida para todos os dados, e que o teste de correlação de Spearman foi utilizado, já que estávamos interessados em algumas correlações em alguns dados categóricos e binários. Análises de covariância (ANCOVA) foram usadas para investigar os efeitos do AUS em pacientes idosos normotensos e hipertensos, com controles para as variáveis de confusão e fatores de risco: idade, sexo e IMC. Da mesma forma, ANCOVA foi considerada para avaliar os efeitos dos níveis de EIM e os níveis de TFG (avaliadas tanto pela fórmula MDRD quanto pela fórmula CKD-EPI) nos níveis de AUS, considerando pacientes idosos com uricemia normal e a hiperuricemia.

A análise estatística foi realizada com um nível de significância de 5%. Foi utilizado o software IBM SPSS Statistics versão 20.0 para Windows. Consideramos estatísticas descritivas, figuras e tabelas para resumir nossos resultados.

## Resultados

Um total de 1920 pacientes adultos (com idade igual ou superior a 18 anos) foram incluídos na análise, dos quais 447 eram pacientes idosos (com idade igual ou superior a 65 anos, 23,28%). A Tabela 1 resume as características de linha de base dos pacientes analisados, e a Tabela 5, as características antropométricas da população.

**Tabela 1:** (*) – Comparação entre os parâmetros estudados de pacientes, baseada em idade (características da linha de base)

	Pacientes adultos (N=1473)	Pacientes idosos (N=447)	p-valor
**Categorias para pressão sanguínea**			
Normotensos	894 (60,69%)	146 (32,66%)	<0,001
Hipertensos	579 (39,31%)	301 (67,34%)	
**Hipertensão - incluindo apenas pacientes hipertensos (#)**			
Controlada	154 (26,60%)	42 (13,95%)	<0,001
Não controlada	425 (73,40%)	259 (86,05%)	
**AUS (mg/dl)**			
N	1473	447	<0,001
Média (DP)	4,89 (1,293)	5,40 (1,479)	
**EIM (mm)**			
N	1059	338	<0,001
Média (DP)	0,60 (0,124)	0,80 (0,140)	
**TFG**_MDRD_			
N	1473	447	<0,001
Média (DP)	85,51 (17,623)	69,36 (18,134)	
**TFG**_CKD-EPI_			
n(%)	1473	447	<0,001
Média (DP)	94,47 (17,347)	69,82 (16,876)	
Hipertensão - incluindo apenas pacientes hipertensos (#)			

* os p-valores são obtidos com testes t de amostras independentes (*) e com testes Qui-quadrado. Os dados contínuos (*) são resumidos como média (pacientes do desvio padrão).

Foi obtida diferença estatística significativa entre a proporção de pacientes hipertensos nos dois grupos estudados. A HT se apresentou com mais frequência no grupo de pacientes idosos (p<0,001). Considerando-se valores de HT controlados, apenas 42 pacientes (13,95%) dos 301 pacientes hipertensos incluídos no grupo dos idosos, parecem ter valores de PA controlados. Houve uma proporção estatisticamente significativa mais alta de pacientes com HT controlada no grupo de adultos, em comparação ao grupo de idosos, considerando apenas pacientes hipertensos (p<0,001).

**Figura 1:**
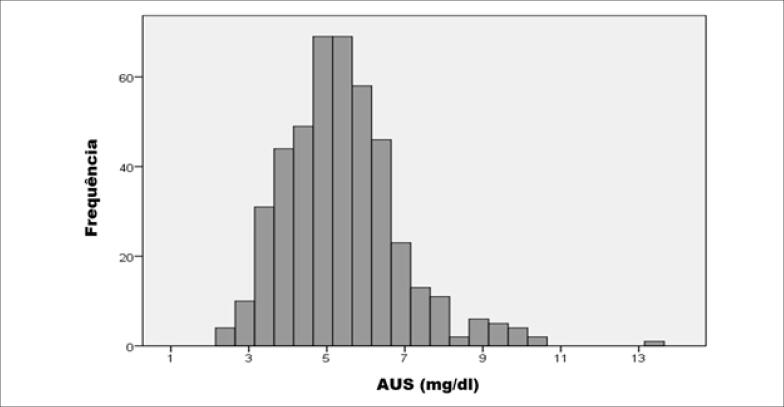
Distribuição dos valores de AUS no grupo de pacientes idosos*. AUS: ácido úrico sérico.

Analisando os valores de AUS, foi obtida uma diferença significativa no valor médio de AUS entre os dois grupos. Pacientes adultos tinham níveis de AUS significativamente mais baixos, em média 0,51 mg/dl, comparados a pacientes idosos (4,89 mg/dl vs. 5,40 mg/dl, p<0.001). (Figura 1)

Ao estudar os níveis de AUS por grupos de pacientes idosos normotensos e hipertensos, os valores mais altos foram observados em pacientes idosos hipertensos, sendo significativamente mais altos em comparação aos registrados para pacientes idosos normotensos. As diferenças permaneceram após padronizar para idade, sexo e IMC (Tabela 2). Pacientes idosos hipertensos, comparados a pacientes idosos normotensos, tinham níveis de AUS significativamente mais baixos, em média 0,39 mg/dl (5,53 mg/dl vs. 5,14 mg/dl, p=0.008).

**Tabela 2:** Ácido úrico sérico por grupos de pacientes idosos normotensos e hipertensos

	Pacientes idosos normotensos (N=146)	Pacientes idosos hipertensos (N=301)	p-valor
Não padronizado	5,14 (0,122)	5,53 (0,085)	0,008
Padronizado para idade	5,13 (0,122)	5,53 (0,085)	0,007
Padronizado para sexo	5,12 (0,118)	5,53 (0,082)	0,005
Padronizado para IMC	5,17 (0,121)	5,51 (0,084)	0,021

* Notas: os p-valores são obtidos com o teste ANCOVA; os valores são resumidos como média (erro padrão). IMC: índice de massa corporal.

Entretanto, os níveis de AUS em pacientes idosos hipertensos não variaram em relação à condição de controle de HT (p=0,632). Apenas 1059 dos 1473 pacientes adultos e 338 dos 447 pacientes idosos tiveram seus valores de EIM medidos. Foi detectada uma diferença significativa no valor médio de EIM, com níveis mais baixos de EIM em pacientes adultos, em média com 0,20 mm, em comparação com pacientes idosos (p<0.001). Ao considerar apenas o grupo dos idosos, não houve diferenças significativas nos valores de EIM, considerando valores de AUS (p=0,510). (Tabela 3)

**Tabela 3:** Ácido úrico sérico por grupos de pacientes idosos normotensos e hipertensos

	Pacientes idosos com uricemia normal (N=356)	Pacientes idosos com hiperuricemia (N=91)	p-valor
Pacientes incluídos na análise (*)	262	76	
Não padronizado	0,80 (0,009)	0,82 (0,016)	0,373
Padronizado para idade	0,80 (0,008)	0,81 (0,015)	0,510
Padronizado para sexo	0,80 (0,009)	0,82 (0,016)	0,119
Padronizado para IMC	0,80 (0,009)	0,82 (0,016)	0,380

* Notas: os p-valores são obtidos com o teste ANCOVA; os valores são resumidos como média (erro padrão). (*) Análises baseadas em pacientes com valores de EIM medidos.

Foi detectada uma diferença significativa no valor médio de TFG_MDRD_; pacientes adultos tiveram níveis significativamente mais altos de TFG_MDRD_, em média com 16,15 mm/min/1,73m^2^, em comparação com pacientes idosos (p<0.001). Os mesmos resultados foram obtidos ao se utilizar a TFG_CKD-EPI_. Pacientes adultos tiveram níveis significativamente mais altos de TFG_CKD-EPI_, em média com 24,65 mm/min/1,73m^2^, em comparação com pacientes idosos (p<0.001) (Tabela 1). Ao considerar apenas o grupo de idosos, os valores mais baixos da taxa de filtração glomerular (TFG) estimada, avaliados pela fórmula MDRD e pela fórmula CKD-EPI, foram observados em pacientes com hiperuricemia, sendo esses valores significativamente mais baixos do que os níveis de TFG registrados em pacientes idosos com uricemia normal. Todas essas diferenças permaneceram estatisticamente significativas após padronizar para idade, sexo e IMC (Tabela 4).

**Tabela 4:** Níveis de ácido úrico sérico e função renal por grupos de pacientes idosos com uricemia normal e hiperuricemia

		Pacientes idosos com uricemia normal (N=356)	Pacientes idosos com hiperuricemia (N=91)	p-valor
TFG_MDRD_				
	Não padronizado	71,50 (0,936)	61,01 (1,851)	<0,001
	Padronizado para idade	71,43 (0,919)	61,25 (1,818)	<0,001
	Padronizado para sexo	71,50 (0,938)	61,00 (1,862)	<0,001
	Padronizado para IMC	71,49 (0,938)	61,05 (1,866)	<0,001
TFG_CKD-EPI_				
	Não padronizado	71,86 (0,869)	61,83 (1,719)	<0,001
	Padronizado para idade	71,78 (0,832)	62,17 (1,646)	<0,001
	Padronizado para sexo	71,92 (0,870)	61,59 (1,727)	<0,001
	Padronizado para IMC	71,91 (0,871)	61,66 (1,733)	<0,001

* Notas: os p-valores são obtidos com o teste ANCOVA; os valores são resumidos como média (erro padrão).

**Tabela 5:** Comparação entre as características principais da linha de base e antropométricas de pacientes adultos e idosos

		Pacientes adultos (N=1473)	Pacientes idosos (N=447)	p-valor
IMC (Kg/m²)(*)				
	n	1473	447	<0,001
	Média (DP)	27,70 (5,892)	29,91 (5,157)	
EIM (mm)				
	n	1059	338	<0,001
	Média (DP)	0,60 (0,124)	0,80 (0,140)	

DP: desvio padrão; IMC: índice de massa corporal.

## Discussão

A HT é uma condição altamente prevalente cuja incidência aumenta dramaticamente com a idade. De acordo com o JNC, a hipertensão ocorre em mais de dois terços dos indivíduos após a idade de 65 anos.^2^ Além disso, dados do Estudo de Framingham, em homens e mulheres sem hipertensão, aos 55 anos de idade, indicam que os riscos de desenvolvimento de hipertensão durante o restante do tempo de vida, até os 80 anos, são de 93% e 91% respectivamente.^7^ Mais de 90% dos indivíduos que não têm hipertensão aos 55 anos de idade vão desenvolvê-la durante o restante de seu tempo de vida. Conforme esperado, a prevalência da HT no grupo dos idosos foi significativamente mais alta.

O efeito da idade no controle de hipertensão ainda parece ser controverso. Um estudo transversal em série das diferenças de idade no controle da HT em consultórios médicos nos EUA, de 2003 a 2010, sugere que pacientes mais velhos tinham probabilidade maior de conseguir fazer o controle da hipertensão em comparação a pacientes mais jovens, assim como relatórios de NAMCS, mas diferentemente da pesquisa National Health and Nutrition Examination Survey.^8^^,^^9^

Os resultados do estudo SEPHAR III demonstraram que os pacientes idosos romenos tinham uma porcentagem reduzida de HT controlada (13,95%), significativamente mais baixa em comparação ao grupo de adultos. O controle abaixo do ideal da hipertensão em pacientes idosos pode estar relacionado ao mal controle, hábitos alimentares, ou uso de um tratamento menos agressivo, com menos medicamentos ou doses mais baixas do que os mais jovens.

Os níveis de AUS estão fortemente correlacionados ao envelhecimento. Os dados do SEPHAR III reconfirmam os valores aumentos de AUS na população >65 anos, e especialmente em pacientes com HT. Como esperado, pacientes idosos apresentaram EIM aumentada. Embora os estudos anteriores tenham demonstrado uma correlação entre os valores de EIM e níveis de AUS, nossa análise, que considerou apenas pacientes com idade >65 anos, não apresentou diferenças significativas na EIM entre os subgrupos de AUS, após a padronização para idade.^7^ Esses resultados são consistentes com os estudos anteriores, sugerindo que o relacionamento entre o AUS e a placa não existia, ou era muito fraco e facilmente influenciável por outros fatores.^10^^,^^11^

A associação entre hiperuricemia e doença renal crônica foi apresentada anteriormente.^12^ Entre os pacientes idosos, os valores de AUS foram significativamente aumentados, independentemente da função renal, o que também se confirmou em um estudo japonês envolvendo mulheres idosas.^13^ Os resultados do estudo SEPHAR III sugerem que a idade e o AUS têm um efeito sinérgico no status da PA, independentemente dos fatores convencionais de risco cardiovascular.

Este estudo tem algumas limitações, tais como o impacto do tratamento contínuo de doenças crônicas nos níveis de ácido úrico sérico. Os pacientes foram questionados sobre seu uso atual de medicamentos e se eles aderiram ao tratamento. Entretanto, medicamentos usados anteriormente não foram documentados. Para se estabelecer uma relação, considerou-se que duas visitas, com um intervalo de vários dias, não foram suficientes para quantificar o impacto dessas intervenções. Além disso, essa análise faz parte de um estudo maior que incluiu adultos com idade igual ou superior a 18 anos, e, portanto, a proporção de pacientes idosos é mais baixa, o que poderia limitar seu poder de caracterização dessa faixa etária.

Documentos recentes sobre hiperuricemia também enfatizaram a associação crescente entre os níveis de ácido úrico sérico e doenças cardiovasculares. O estudo Uric Acid Right for Heart Health (URRAH) envolvendo mais de 22000 sujeitos mostraram demonstrou, por meio de análises de regressão multivariadas de Cox, que o ácido úrico sérico é um fator de risco de mortalidade independente.^14^

Outros estudos avaliaram o efeito do ácido úrico sérico na rigidez arterial em pacientes hipertensos e não identificaram influência da progressão da velocidade da onda de pulso na população estudada, depois de um acompanhamento médio de 3,8 anos. Os autores desse estudo^15^avaliaram 422 pacientes hipertensos adultos apresentaram, em uma população não padronizada, associação significativa entre rigidez de vasos e ácido úrico sérico, mas a significância foi perdida quando houve a padronização para parâmetros diferentes, como, por exemplo, índice de massa corporal.

Uma análise diferente sobre a Europa central e oriental também demonstrou um aumento na prevalência da hiperuricemia em pacientes hipertensos, com pelo menos um quarto da população estudada apresentando níveis aumentados de ácido úrico sérico. Na análise covariada de variáveis cardionefrometabólicas dos 3206 pacientes do estudo BP-CARE, a única relação significativa entre os níveis de ácido úrico sérico encontrada foi com doença renal crônica.^16^

Além disso, há vários outros estudos que demonstram uma ligação entre os níveis de AUS e outros parâmetros metabólicos, tais como colesterol LDL, evidenciando uma relação entre esses dois em relação ao risco de desenvolvimento de hipertensões nas etapas mais tardias da vida.^17^ No caso dos idosos, há outros estudos que corroboram o achado de que o AUS geralmente é encontrado na síndrome metabólica, tais como o relatório dos autores do Estudo Cardíaco de Brisighella.^18^ Em nossa análise, encontramos diferenças significativas entre pacientes idosos e adultos, sendo que os pacientes idosos são mais obesos e têm uma EIM mais alta.

Ainda existe um debate para se definir se o ácido úrico sérico tem um efeito pequeno na rigidez dos vasos, tem um efeito sinérgico com outro fator de risco, ou não tem efeito nenhum. Entretanto, a hiperuricemia deve ser tratada ainda assim.

## Conclusão

Este estudo é o primeiro que apresenta dados específicos dos valores de HT e AUS focados em pacientes idosos romenos. Embora cada vez seja mais reconhecido que a idade biológica é mais importante que a idade cronológica, o tratamento e o controle de HT na população mais velha devem ser otimizados, considerando características individuais de saúde, já que o tratamento reduz mortalidade, acidente vascular cerebral e insuficiência cardíaca. Este estudo destaca que os níveis de AUS aumentados estão associados ao envelhecimento, e as correlações com a HT foram identificadas, independentemente do estado da função renal.
