# Supplementary material for: Hipertensão Arterial e Ácido Úrico Sérico em Idosos - Estudo SEPHAR III
Source: Arq Bras Cardiol. 2021 Aug 9;117(2):378–84. [Article in Portuguese] doi: 10.36660/abc.20200004 (PMC8395801; doi:10.36660/abc.20200004)
Supplement: Translation

# Translation: Arterial Hypertension and Serum Uric Acid in Elderly- SEPHAR III Study

**Authors:** Roxana Buzas, Vlad-Sabin Ivan, Oana-Florentina Gheorghe-Fronea, Adina Flavia Morgovan, Melania Ardelean, Nicolae Albulescu, Maria Dorobantu, Daniel Florin Lighezan

**Affiliations:** 1 Timisoara Romania University of Medicine and Pharmacy Victor Babes Timisoara - Department of Internal Medicine, Timisoara – Romania.; 2 Bucuresti Romania Carol Davila University of Medicine and Pharmacy - Cardio-Thoracic Pathology Department, Bucuresti – Romania.

**Keywords:** Hypertension, Uric Acid, HYperuricemia, C-Effectardiovascular Diseases, Glomerular Filtration, Age-Effect

## Abstract

**Background:**

Hyperuricemia is a frequent finding in patients with arterial hypertension, and there is increasing evidence that this entity is also a risk factor for cardiovascular disease.

**Objective:**

In the context of an aging population, this study aims to evaluate serum uric acid levels and arterial hypertension prevalence and control in a subgroup of Romanian adults (>65 years), concerning the influence of age on these parameters.

**Method:**

The study sample consists of 1,920 adults included in SEPHAR III survey, of whom 447 were elderly patients (>65 years of age). During the two study visits, three blood pressure (BP) measurements were performed at 1-min intervals and serum uric acid levels, kidney function by estimated glomerular filtration rate, blood pressure, and intima media thickness measurements were conducted. Hypertension and controls were defined according to the current guidelines. Intima-media thickness evaluation was assessed by B-mode Doppler ultrasound evaluation. A significance level p < 0.05 was adopted for the statistical analysis.

**Results:**

Adult patients had a significant lower serum uric acid levels, compared to elderly patients, regardless of glomerular filtration rate levels. Adult patients showed a significantly lower intima-media thickness levels, when compared to elderly patients.

**Conclusion:**

Similar to previous studies, in the present study, age represented one of the factors contributing to the increased level of serum uric acid. An increasing prevalence of arterial hypertension with age, together with a poor control of blood pressure, was also obtained.

## Introduction

Life expectancy continues to increase in developed countries worldwide, leading to an ever-increasing representation of older adults (people over 65 years of age) within the population.^1^

According to the Eight Report of the Joint National Committee (JNC 8), approximately 970 million people worldwide have high blood pressure. It is estimated that by 2025, 1.56 billion adults will be living with arterial hypertension (HT). The etiology of essential HT still remains unknown; its pathogenesis includes multiple genetic and environmental factors. More than two-thirds of individuals over 65 years of age suffer from HT, according to the Seventh Report of the Joint National Committee (JNC-7).^2^ Several epidemiological studies indicated that the incidence of HT and related cardiovascular disease is higher in the elderly than in the young population.^2^^,^^3^ A study on its prevalence and control among United States adults from 1999 to 2004 showed that the prevalence of HT has more than doubled in the elderly than in the young population. Even if the general belief is that HT is an aging disorder, in recent years, the middle-aged population has shown an increase in the incidence of arterial hypertension.

On the other hand, hyperuricemia is more common, and several studies show that serum uric acid levels are linked to an increase in the prevalence of hypertension (HT), which also contributes to a lack of optimal blood pressure (BP) control.^4^

SEPHAR (Study for the Evaluation of Prevalence of Hypertension and Cardiovascular Risk in Romania) is a project that aims to evaluate the prevalence of HT and other related factors including serum uric acid. To date, three separate SEPHAR studies have been conducted at several years intervals, with SEPHAR II being conducted in 2012, which was the first to evaluate the serum uric acid levels, which also correlated the serum uric acid (SUA) levels with intima media thickness, renal function, and cardiovascular risk. Continuing with SEPHAR III in 2016, which provided further data on SUA levels and its relationship with HT prevalence in Romania, several other indices were also used, such as eGFR and echocardiographic parameters. SEPHAR III was designed as a cross-sectional survey for characterizing data for the adult population in Romania for HT prevalence, control, and antihypertensive agents.^5^^,^^6^

This paper aims to evaluate SUA levels, IMT and HT prevalence, and control, in a group of Romanian adults, concerning the aging population.

## Material and methods

A mobile medical caravan dubbed SEPHAR Bus was used to perform two visits, at a 4-day interval between them. Overall, 1,920 Romanian adults were enrolled in this SEPHAR III survey (mean age 48.63 years, 52.76% females), of whom 447 were elderly patients (23.28%, 65 years of age or older). Patients were examined and three BP measurements, in accordance with the current European Guidelines for BP monitoring, were performed at one-minute intervals while sitting. During each visit, three sitting BP measurements, with an automated BP measurement device (OMRON M6), were registered. The cuff was adjusted for the arm’s circumference, and all of the measurements were performed on the same arm that presented the highest BP values during the inaugural visit.

A systolic blood pressure (SBP) of more than 140mmHg and/or diastolic blood pressure (DBP) greater than 90mmHg in both visits was considered HT, using the average of the second and third BP values of each visit. The first BP of each visit was not taken into consideration for further analysis. Moreover, known and treated HT, with controlled or uncontrolled BP during the previous two weeks, was also taken into consideration.

For a subject to have controlled BP, 2018 ESH-ESC guidelines on hypertension was used, defining a BP control for hypertensive subjects with at least two weeks of prior treatment, an SBP and a DBP of less than 140mmHg and 90mmHg, respectively.

Blood sample analysis that included the aforementioned SUA was performed during the second visit, with the patient being informed in the first visit that a fasting period of at least 8 hours would be required. SUA levels were analyzed with a COBAS 6000 analyzer with uricase/peroxidase reagents, with normal values given between 2.4 to 5.70mg/dl in females and 3.40 to 7.00mg/dl in males. Hyperuricemia was diagnosed when above normal ranges were identified. For the evaluation of the kidney function, both Modification of Diet in Renal Disease formula (MDRD) and Chronic Kidney Disease Epidemiology Collaboration formula (CKD-EPI) values were calculated and used in the statistical analysis.

A portable echocardiograph (model General Electric Vivid Q), which automatically calculated the intima-media thickness (IMT) of each distal wall of the common carotid artery, 1 cm below the carotid bulb, was used. The IMT was measured using a linear probe with an adjustable frequency between 7.5 and 10 MHz.

The Ethics Committee of the “Carol Davila” University of Medicine and Pharmacy, Bucharest, Romania, approved the study in complete accordance with the Declaration of Helsinki and written consent was required from all participants before any examination was undertaken.

### Statistical analysis

Results for targeted variables were presented, using counts with corresponding percentages for categorical data and descriptive statistics (mean, standard deviation) for continuous data. Differences in means for continuous variables were analyzed using t-tests for independent samples, while Chi-squared test was used to examine differences between categorical variables. Considering the sample size normality was assumed for all data, and the Spearman correlation test was used, as we were interested in some correlations in some categorical and binary data. Analyses of covariance (ANCOVA) were used to investigate the effects of SUA on normotensive and hypertensive elderly patients, with controls for the confounding variables and risk factors: age, gender, and BMI. Similarly, ANCOVA was considered to assess the effect of IMT levels and eGFR levels (assessed both by MDRD and by CKD-EPI formula) on SUA levels considering normouricemia and hyperuricemia elderly patients.

Statistical analysis was performed with a significance level of 5%. The IBM SPSS Statistics, version 20.0, software for Windows was used. Descriptive statistics, figures, and tables were considered to summarize our results.

## Results

A total of 1,920 adult patients (18 years of age or older) were included in the analysis, of whom 447 were elderly patients (65 years of age or older, 23.28%). Table 1 summarizes baseline characteristics of the analyzed patients, and Table 5 summarizes the baseline anthropometric characteristics of the population.

**Tabela 1:** (*) – Comparison between studied parameters of patients based on age (baseline characteristics)

	Adult Patients (N=1473)	Elderly Patients (N=447)	p-Value
**Categories for blood pressure**			
Normotensive	894 (60.69%)	146 (32.66%)	<0.001
Hypertensive	579 (39.31%)	301 (67.34%)	
**Hypertension – including only hypertensive patients (#)**			
Under control	154 (26.60%)	42 (13.95%)	<0.001
Not under control	425 (73.40%)	259 (86.05%)	
**SUA (mg/dl)**			
N	1473	447	<0.001
Mean (SD)	4.89 (1.293)	5.40 (1.479)	
**IMT (mm)**			
N	1059	338	<0.001
Mean (SD)	0.60 (0.124)	0.80 (0.140)	
**IMT (mm)**			
N	1473	447	<0.001
Mean (SD)	85.51 (17.623)	69.36 (18.134)	
**eGFR**_MDRD_			
n	1473	447	<0.001
Mean (SD)	94.47 (17.347)	69.82 (16.876)	

* p-values are obtained with independent sample t-tests (*) and with Chi-square tests. Continuous data (*) are summarized as mean (standard deviation patients).

**Tabela 5:** Comparison between main baseline and anthropometric characteristics between adult and elderly patients

		Adult patients (N=1473)	Elderly patients (N=447)	p-value
BMI (kg/m^2^)(*)				
	n	1473	447	<0.001
	Mean (SD)	27.70 (5.892)	29.91 (5.157)	
IMT (mm)				
	n	1059	338	<0.001
	Mean (SD)	0.60 (0.124)	0.80 (0.140)	

SD: standard deviation; BMI: body mass index.

Significant statistical difference was found among the proportion of hypertensive patients in the two studied groups. HT was more frequent in the elderly group (p<0.001). Considering controlled HT values, only 42 patients (13.95%) of the 301 hypertensive patients included in the elderly group seem to have controlled BP values. A significantly statistical higher proportion of patients with controlled HT was identified in the adult group when compared to the elderly group, considering only hypertensive patients (p<0.001).

Analyzing the SUA values, a significant difference was obtained in the mean value of SUA in the two groups. Adult patients presented significantly lower SUA levels, on average, with 0.51mg/dl, as compared to elderly patients (4.89 mg/dl vs. 5.40mg/dl, p<0.001).(Figure 1)

**Figure 1:** Distribution of the SUA values in the elderly patients’ group*. SUA- serum uric acid.

When studying SUA levels by groups of normotensive and hypertensive elderly patients, the highest values were observed in hypertensive elderly patients, these values being significantly higher when compared to those recorded in normotensive elderly patients. The differences remained after adjusting for age, sex, and BMI (Table 2). Hypertensive elderly patients compared to normotensive elderly patients had significantly higher SUA levels, on average, with 0.39 mg/do (5.53 mg/dL vs. 5.14 mg/dL, p=0.008).

**Tabela 2:** Serum uric acid by groups of normotensive and hypertensive elderly patients

	Normotensive Elderly Patients (N=146)	Hypertensive Elderly Patients (N=301)	p-Value
Unadjusted	5.14 (0.122)	5.53 (0.085)	0.008
Adjusted for age	5.13 (0.122)	5.53 (0.085)	0.007
Adjusted for gender	5.12 (0.118)	5.53 (0.082)	0.005
Adjusted for BMI	5.17 (0.121)	5.51 (0.084)	0.021

* Notes: p-values are obtained with ANCOVA test; values are summarized as mean (standard error). BMI: body mass index.

However, SUA levels in hypertensive elderly patients did not change regarding the HT control status, p=0.632). Only 1,059 of the 1,473 adult patients and 338 of the 447 elderly patients had their IMT values measured. A significant difference in mean value of IMT was obtained with lower IMT levels in adult patients, on average, with 0.20mm, as compared to elderly patients p<0.001). When considering only the elderly group, no significant differences were found in IMT values when considering SUA levels (p=0.510)(Table 3)

**Tabela 3:** Serum uric acid by groups of normotensive and hypertensive elderly patients

	Normouricemia Elderly Patients (N=356)	Hyperuricemia Elderly Patients (N=91)	p-Value
Patients included in the analysis (*)	262	76	
Unadjusted	0.80 (0.009)	0.82 (0.016)	0.373
Adjusted for age	0.80 (0.008)	0.81 (0.015)	0.510
Adjusted for gender	0.80 (0.009)	0.82 (0.016)	0.119
Adjusted for BMI	0.80 (0.009)	0.82 (0.016)	0.380

* Notes p-values are obtained with ANCOVA test, values are summarized as mean (standard error). (*) Analyses based on patients with measured IMT values.

Significant differences in the mean value of eGFR_MDRD_ were obtained; adult patients presented significantly higher eGFR_MDRD_levels, on average, with 16.15 ml/min/1.73m^2^, as compared to elderly patients (p<0.001). The same results were obtained when using eGFR_CKD-EPI_. Adult patients presented significant higher eGFR_CKD-EPI_ levels, on average, with 24.65 ml/min/1.73m^2^, as compared to elderly patients (p<0.001) (Table 1). When considering only the elderly group, the lower values of the estimated glomerular filtration rate (eGFR), assessed by both MDRD and CKD-EPI formulas, were observed in elderly patients with hyperuricemia, with these values being significantly lower than eGFR levels recorded in elderly patients with normouricemia. All of these differences remained statistically significant after adjusting for age, sex, and BMI (Table 4).

**Tabela 4:** Serum uric acid levels and renal function by groups of normouricemia and hyperuricemia elderly patients

		Normouricemia Elderly Patients (N=356)	Hyperuricemia Elderly Patients (N=91)	p-Value
**eGRF_MDRD_**				
	Unadjusted	71.50 (0.936)	61.01 (1.851)	<0.001
	Adjusted for age	71.43 (0.919)	61.25 (1.818)	<0.001
	Adjusted for gender	71.50 (0.938)	61.00 (1.862)	<0.001
	Adjusted for BMI	71.49 (0.938)	61.05 (1.866)	<0.001
**eGRF_CKD-EPI_**				
	Unadjusted	71.86 (0.869)	61.83 (1.719)	<0.001
	Adjusted for age	71.78 (0.832)	62.17 (1.646)	<0.001
	Adjusted for gender	71.92 (0.870)	61.59 (1.727)	<0.001
	Adjusted for BMI	71.91 (0.871)	61.66 (1.733)	<0.001

* Notes: p-values are obtained with ANCOVA test, values are summarized as mean (standard error).

## Discussion

HT is a highly prevalent condition that dramatically rises in incidence with increasing age. According to JNC, hypertension occurs in more than two-thirds of individuals after 65 years of age.^2^ Moreover, data from the Framingham Heart Study, in men and women free of hypertension at 55 years of age, indicate that the remaining lifetime risks for development of hypertension through 80 years of age are 93% and 91%, respectively.^7^ More than 90% of all individuals who are free of hypertension at 55 years of age will develop it during their remaining lifespan. As expected, the prevalence of HT in the elderly group was significantly higher.

The effect of age on hypertension control still seems to be controversial. A Serial Cross-sectional study of age differences in the control of HT in US Physician’s Offices, from 2003-2010, suggests that older patients were more likely to achieve hypertension control when compared to younger patients, which is the same as findings from NAMCS but in contrast with the National Health and Nutrition Examination Survey.^8^^,^^9^

SEPHAR III results revealed that elderly Romanian patients have a reduced percentage of controlled HT (13.95%) that is significantly lower when compared to the adult group. Suboptimal hypertension control in older patients may be related to poor management, culinary habits, or less aggressive treatment, using, with fewer medications or lower doses than their younger counterparts.

SUA levels are strongly correlated with aging. SEPHAR III data reconfirms SUA increased values in the population of >65 years and especially in HT patients. As expected, elderly patients had an increased IMT. Although previous studies showed a correlation between IMT values and SUA levels, our analysis, which considered only patients aged >65 years, revealed no significant differences in IMT among SUA subgroups after adjusting for age.^7^ These results are consistent with previous studies suggesting that the relationship between SUA and plaque was nonexistent or very weak and easily influenced by other factors.^10^^,^^11^

The association between hyperuricemia and chronic kidney disease was presented above.^12^ Among elderly patients, SUA values were significantly increased, regardless of renal function, which is the same as data from a Japanese study with elderly women.^13^ SEPHAR III results suggest that age and SUA have a synergistic effect on BP status, regardless of conventional cardiovascular risk factors.

The present study has some limitations, such as the impact of ongoing treatment for chronic diseases on the levels of serum uric acid. The patients were questioned on their current medications and whether they are adherent to therapy, but earlier medications were not documented. To establish such a relationship, we consider that two visits, with intervals of several days between them, were not enough to quantify the impact of such interventions. This analysis is also part of a larger study that encompassed adults of 18 years of age or over; therefore, the proportion of elderly patients is lower, which could limit its power to characterize this age group.

Recent papers on the risk of hyperuricemia have also stressed the increased association between the levels of serum uric acid and cardiovascular disease. The Uric Acid Right for Heart Health (URRAH) study of over 22,000 subjects showed, through multivariate Cox regression analyses, that the serum uric acid is an independent risk factor for mortality.^14^

Other studies evaluated the effect of serum uric acid on arterial stiffness in hypertensive patients and found no influence on the progression of pulse wave velocity in the studied population after a median follow-up of 3.8 years. The authors of this study^15^ evaluated 422 adult hypertensive patients and showed, in an unadjusted population, significant association between vessel rigidity and serum uric acid, but the significance was lost when adjusted for different parameters for example such as BMI.

A different analysis regarding Central and Eastern Europe has also shown an increased prevalence of hyperuricemia in hypertensive patients with at least one quarter of the studied population having increased levels of serum uric acid. In the covariate analysis with cardionephrometabolic variables, of the 3,206 patients from the BP-CARE study, the only significant relationship between serum uric acid levels was found to be with chronic kidney disease.^16^

There are also several other studies that show a link between the levels of SUA and other metabolic parameters, such as LDL-cholesterol, showing a relationship between these two as regards the risk of developing hypertensions in the latter stages of life.^17^ In the elderly, there are other studies that support the finding that SUA is often found in metabolic syndrome, such as the report from the authors of Brisighella Heart Study.^18^ In our analysis, a significant difference was found between the elderly versus adult patients, with elderly patients being more obese and having a higher IMT.

Whether the serum uric acid has a minor effect on vessel rigidity, acts synergistically with other risk factor, or has no effect at all is still a debate that needs to be answered, but hyperuricemia should be treated nonetheless.

## Conclusion

Our study is the first of its kind that provides specific data of HT and SUA values focused on Romanian elderly patients. Although it is increasingly recognized that biological rather than chronological age is important, HT treatment and control in older populations must be optimized, considering individual health characteristics, since therapy reduces mortality, stroke, and heart failure. Our study serves to emphasize that increased SUA levels are associated with aging and correlations with HT are identified, regardless of the state of renal function.
